# Proceedings of the Maryland and District of Columbia Dental Society

**Published:** 1880-01

**Authors:** 


### ARTICLE IV.

[Continued from last Number.]

### *Proceedings of the Maryland and District of Columbia Dental Society. Annual Meeting.*

We feel stronger each year in the opinion that a dental college should be free from all entangling alliances in the way of medical schools. Having sent students to and had assistants from both the pure blood and the half-breed schools, my opinion is that the instruction in the pure dental college is superior in point of such medical knowledge as the dentist needs, and why? Simply because it is taught from a dental stand-point and he is given the particular instruction he needs, and the mind is not overtaxed or burdened with the details and treatment of a thousand diseases not at all essential for a dentist to know, as it would be if he was called upon to treat them. If any dentist or student has the time and the means, and desires to master all the details of disease or the functions of each muscle and bone, let him attend a medical school and take the degree of M. D., but the title of M. D., does not show that he has received instruction in, or is competent to practice dentistry; but the title of D. D. S., does show that he has been so instructed. Let us not confound dentistry with medicine; any attempt to mix the two professions will weaken both; let us walk together as brothers having interests and studies in common; thus can we best assist each other in relieving suffering humanity.

We would again direct attention to the many valuable ldeas and records of actual practice and experiments that are printed in our journals from year to year, but are soon forgotten or lost to the dental student by being buried in a book-case or moulding in some back closet.

These records and experiments ought to be collected and tabulated; all new and interesting cases be noted and put in such shape that they could be made useful to all dental students, and when we say students we do not mean alone those entering or seeking to enter our profession, but all seekers of knowledge.

Our journals are constantly publishing things as new that are old and well known to the profession, such a collated tabulated or epitomized dental history.

It is to be hoped that some of the younger members of our profession will see the need and be stimulated to take hold of such a work, that every society and every well-posted dentist needs; not an essay or address is prepared but the need of such a work is felt unless he is a walking encyclopedia.

Such a work would keep us from being annoyed by many of the patents gotten out perhaps through ignorance of what had been in use both by the inventor and patent examiner.

The minds of our young men should be directed to the scientific aspects of our profession. The microscope and its use should be carefully studied so that we may know and be familiar with its use in studying the structure and disease of the dental organs. When we are so instructed there will be little trouble or thought of our professional standing.

The paper being open for discussion, Dr. Coy said he approved the whole paper, particularly the points of preliminary examination.

Dr. Caldwell thought there was too much strictness in examination for graduation, saying that although candidates might fail on examination they should still have a chance.

Dr. Winder said he had always been in favor of preliminary examinations, the standard not being too high or too low but such as would give a good foundation for professional education. That every diploma in the world should be granted because the candidate deserved it. He was in favor of a board of examiners, as tending to secure more impartiality in the examinations and consequently more weight and worth to the diploma.

Dr. Cutter approved of having a professional man thoroughly educated.

After a recess, the discussion of the morning was continued by Dr. E. P. Keech, who fully endorsed the position taken by Dr. Winder and made extended remarks advocating state legislations regulating the practice of dentistry for the protection of the profession and the public.

On motion of Dr. McPherson, the order of business was suspended to enable the Executive Committee to bring forward at this meeting the subject of printing the proceedings. Dr. Gorgas and Mr. Cowman were present, and after consulting together, through Dr. Gorgas, proposed that they would publish the proceedings in the American Journal of Dental Science, if they could do so in consecutive numbers, so as not to take up too much of any one number. The Association accepted this liberal proposition and tendered thanks for it.

The subject of Dental legislation was resumed, and after full expression of views on the subject by Drs. E. P. Keech, Winder, Gorgas, Foster, Coy, Hunt, and Atkinson, Dr. E. P. Keech offered the following resolution which was adopted:

*Resolved*, That a Committee of three, of which the President of this Association shall be ex-officio, be elected with power to prepare and submit to the Legislature, a bill for the protection of the practice of Dentistry in the State of Maryland.

Dr. Winder moved that the Committee be instructed that the Association wishes a law passed that will require an examination and a diploma, and that no one can be examined unless he is in possession of a diploma. Adopted.

Dr. Noble read a letter from Dr. B. M. Wilkerson, tendering his resignation as a member of this Society, on account of the action of the Association with reference to the White Dental Chair. The resignation was accepted.

On motion the Association then proceeded to the election of officers with the following result:

*President.*—Dr. M. Whilldin Foster, - Md.
*Vice-President.*—H. B. Noble, - - - D. C.
*Rec. Secretary.*—H. M. Schooley, - - D. C.
*Rep. Secretary.*—J. B. Ten Eyck, - - D. C.
*Treasurer.*—T. W. Coyle, - - - - - Md.

*Executive Committee.*—Drs. R. B. Donaldson, J. L. Wolf, C. E. Duck.

*Committee on Legislation.*—Drs. M. W. Foster, *Chairman ex-officio*; R. B. Winder, B. F. Coy, T. S. Waters.

Drs. B. F. Coy, and T. W. Coyle were appointed to conduct the newly elected President and Vice-President to their chairs, who took their stations with a few appropriate remarks.

On motion of Dr. Winder, the name of Dr. E. P. Keech was by a unanimous vote added to the Legislative Committee.

The regular order of business was postponed to hear Dr. Ephraim Cutter, of Boston, read an article on "Food," as follows:

"FOOD IS AN AGENT OF TREMENDOUS POWER."—Dr. J. H. Salisbury, Cleveland, Ohio.

One of the finest traits of human character is the love of the beautiful. Harmonies charm our ears. The beauties revealed by the use of the microscope delight our eyes. Perfumes regale our sense of smell. When sick, the touch of the nurse's hand soothes our aching limbs. Food delightfully sates our taste. Especially when the sense of hunger takes possession, does it seem as if nothing could be more beautiful than meats and drinks. This brings us to allude to the history of Food as an *Aesthetic Power.* Music may thrill us. The rapt observer may forget all in gazing at the forms of life found in common pools. The odor of the sea may agreeably brace the enervated frame. Massage may confer a satisfying sense of relief by the frictions of the terminal loops of the sensory nerves. Delicate morsels may cause the gourmand to sink all consciousness in the æsthetics of the palate—but is it not wonderful that food conveys impression to us through all the five sense channels?

The alimentary historian finds that mankind ask in relation to food:

1.—Does it taste nicely?
2.—Does it look goodly?
3.—Does it smell nicely?
4.—Does it affect the hearing nicely?
5.—Does it have a nice impression on the sense of touch?

These queries belong to the domain of æsthetics, that is, they include the idea of the beautiful. For example: Fruit that looks badly, smells badly, sounds uncrisply and has a punky touch, at once produces an impression opposed to the beautiful, and ordinarily is rejected as food.

Note how the æsthetics of the eye are consulted in relation to food. Take a State banquet as one of the highest expressions of food influence, backed by the riches, culture and taste of the Commonwealth. Eating is the central object in the occasion, but the banquet hall is paved with variegated marbles, decorated with plants, cut or whole; adorned with all the skill of the frescoer, painter, artist and upholsterer, so that above the head, beneath the feet, and on every hand the eye is regaled with the beautiful fixtures. The treasures of the loom, the spotless laundering, the marvels of glassery, the products of the most skillful gold and silversmiths, the finest table cutlery, the treasures of porcelain, the sparkling liquors, are spread artistically on heavy tables, rich with massiveness and exquisite carving. Then the edibles themselves in the varied courses, are served up in the most beautiful, sometimes fantastic shapes and forms by attendants whose looks scrupulously avoid anything that offends the eye. The guests themselves are attired after the requirements of the best ocular taste. Indeed, pen cannot do justice to the dress and toilets of the fairest of the fair, to say nothing of the daughters themselves, whom all declare the most beautiful objects found in nature, whom all delight to honor, and without whose presence to grace the occasion, the banquet would be a failure. The animated scene is like "apples of gold set in pictures of silver;" and so in a first-class effort to eat food we find combined in one array a grand display to captivate the sense of sight. On the other hand anything even harmless in itself that offends the eye even at an ordinary meal is enough to drive away the guests. We know of some very worthy practical and common-sense sort of folks who were driven away from a sea-side boarding-house by the fact that the table was covered with variegated enamel cloth. They wanted linen, as they were used to it.

Note the æsthetics of Sound in connection with a State banquet. Skilled artists load the air with chamber music. Sometimes the sounds of a miniature water-fall lull the ear. Then the sweeet music of the voices of loved ones, honored ones, celebrated ones, excite the pleasures of the tympanum. The pleasant noise of knives and forks, the clash of plates, the agreeable jingle of ice in water pitchers; the soft tread of the attendants; the hum of edible industry, all affect the ear delightfully.—(If you doubt, recall your history when an excluded boy you hung outside some civic or military dinner, hearing, seeing, smelling—everything but touching and tasting the forbidden feast.)

What more appetizing and delightful than the sounds of the crackling bubbles of fat in cooking to a hungry fisherman just in from a hard day's sport? The sense of smell seems to have an especial field of exercise in food matters. Delicious odors convey to the sensorium intelligence of the presence of food sooner than sight, sound, or touch. The invisible particles of perfume from fruits in the subtle and unanalyzed alcohols and ethers, volatilized in the air, excite the pleasure of the olfactory nerves—in what manner we know not. They pervade the kitchen, dining-room and house, notifying the waiting guests of the stages and readiness of the repast and adding zest to the appetite. So also disagreeable odors from food command instant recognition sometimes of noxiousness and warning. Often the air of the State banquet halls is loaded with the odors of orange blossoms or delicate colognes. Not but that poisonous aliments may be beautiful to the smell, but generally to most sensoriums, enjoyable odors are foretastes of the gustatory delight.

If any think there are no æsthetics of the sense of touch in the matter of foods, let a gravel-stone get in between the teeth while eating—how the prandal pleasure is marred. "The nerves are set on end," as we say, and not an agreeable "end" either. Or suppose one finds a hair in the breakfast butter or hash—but no need of this disagreeable side of our subject. We want our fruits when touched by the hand to have a certain standard of solidity, else they fail of the requirement and at once are pronounced not good. The standard varies for different articles. We like hardness in butter, but not in melons; toughness in steaks, and other meats is not very beautiful, while tenderness and fragility are prized. We judge of the proper completion of the stages of cooking by the sense of touch. Hence the cook pierces the flesh or vegetable with forks to ascertain the completion of the process. So also we test apples, and fruits, punch peaches and melons, shake cocoa nuts as a *physical exploration* of the exact edible condition of the article in question. It seems then to the writer that he is right in saying that the æsthetics of touch form one of the standards whereby food is judged.

It needs but a mere mention to remind how much the æsthetics of taste judge the fitness of food. To be sure abuse may pervert the gustatory sense, so that often it is warped far away from its natural standard. Some delights of taste are matters of education and culture. We have to learn to eat olives for example. Climate also has to do with tastes. The Esquimaux quaff oil as the Cuban eats pepper. A little travel shows the variation in tastes for food. Still, in health the acts of eating are usually pleasurable to the gustatory nerve, and the love of the beautiful of this sense is gratified; not but that healthy persons eat from the sense of duty, still those who eat for the sake of pleasure probably do not realize the æsthetic gratification of those who eat to live and do not live to eat. When the gustatory nerve gives information as to an article of food of non-æsthetic nature it will be rejected unless stern necessity or famine force. So when excess in eating has cloyed—that is undoubtedly enough—the substance that was longed for and needed now by too much use excites displeasure if not disgust. But often food that is known to be injurious is partaken of because it is æsthetic to the taste—the eater ignoring the certain subsequent misery. Drinks are food. Some are known to drink to drunkenness when they realize the injury of the proceedings. This seems to be a species of insanity as to a given sense.

When it is remembered that food has many other relations than those pertaining to the senses—it seems singular that so one-sided and important a matter is judged so much by the æsthetic standard. But this being natural and instinctive, we say naught against it. We would rather affirm the reasonableness of combining with it other standards. As man has advanced from the savage to the civilized state he has became a recording animal, and thus has acquired a literature of science and art. Workers in past ages have recorded the results of their thoughts and labors, and our present state of knowledge thus derived embraces in relation to food a much larger range of standpoints of view than the purely æsthetical, however practical and important. These deserve to be more widely known and it is desired to call attention to them.

### Food a Power as a Chemic.

Though much remains to be learned and is uncertain in Chemistry, still large additions to our ideas of food comes from this field of human knowledge. The Chemist tells us that our bodies are made up of about 16 (sixteen) ultimate elements, more or less. In his view man's body is an assemblage of so many atoms of Carbon, Hydrogen, Oxygen, Nitrogen, Phosphorous, Sulphur, Lime, Potash, Soda, Iron, Manganese, Magnesia, Iodine, Silica, Boron, Lithium. Some chemists say there are more than forty ultimate elements but others regard them as accidental. Now on this view all our combined food must have all these sixteen elements. On this ground air is food, as it contains Oxygen and Nitrogen. Indeed the fact that life can be sustained for days without eating, while five minutes without breathing results in death seems to settle the question of air as food. Now if we ask the Chemist what is good food, he would answer, those aliments which have passed the criterions of æsthetics and contain in proper combination and in assimilable forms the greater part of the elements found in the human body.

On this view alone, that is if every part is eaten, can cannibalism be justified? Fish thrive on fish. Birds prey on birds. Thus eating they obtain good chemical food. As however man in cannibalism eats muscles, it is difficult to see how bone for example can be organized out of such food. Or if he eats bone how it can be digested, as he does not have the grinding apparatus of the common fowl. Of course should the Chemist portion out of his laboratory the various elements in the proportion found by his analysis and then set a child to eat them, the experiment would not be a success, because the gauntlet of digestion could not be run. But we can ask what natural aliments are bone food, nerve food, blood food, &c. He would say that aliment containing the proper amount of mineral elements in assimilable forms would be good bone food; for example: beans—meals of the five royal grains; Wheat, Rye, Barley, Oats and Maize. The late Dr. Benjamin Cutter, of Woburn, Mass., used to give his surgical cases eggs, including shells, for bone food in cases of fractures and with good results.

So the Chemist tells us butter is good nerve food, as it largely contains fat acids almost identical with those found in nerve tissues. For the blood he states that milk is a perfect food. All body elements are found in it. It has water enough for a drink. It is in this respect uniform. It is also one of the great blessings of the age that "our land flows with milk." But greed has arrived at this superb food and adulteration in brain food for the sake of gain have made it a source of disease. (Dr. R. W. Piper's report Chicago, 1879.) Eggs come next to milk as a Chemical Nerve Food, and the grains, the Legumens, Rice, &c., are good blood food from this point of view. The great Liebig indicated the loss inflicted by altering food from the standard set by the Creator. He alluded especially to the withdrawal of the phosphates and other salts from wheat in the manufacture of flour. The gluten cells that contain the phosphates are rich in the inner layer of the tegumentary cellulose that surrounds the starch substance, and contains the coloring matter; the retention of the gluten cells causes the bread to have a brown, bronze or black color. Society on æsthetic grounds has ordained that flour bread to be good must be white. Hence it follows that there is a withdrawal of 75 per cent. of the mineral matter found in wheat. The Chemist's view is that this large withdrawal is an evil. The writer thinks that the present prevalence of decayed teeth, weak nervous systems, change in the type of disease from strong to weak, premature baldness and gangrene of the hair, the decline in the natural normal increase of the population can be traced to the general use of flour—an impoverished food. No diatribes or hobbyisms are intended, but the solid foundation for this supposition rests on the three-fourths withdrawal of Phosphorus, Lime, Potash, Soda, &c., and on the fact that in cases where the dicta of the Chemist have been listened to and meals have been used in place of flour that there has been a manifest improvement in the respects cited. Thanks to the industry of the Chemist. Almost every vegetable has been analysed, so that those wishing to test food by the chemical standard can readily do so. Prof. Johnson's admissible and valuable work, "How Crops Grow," contains in the appendix many analysis of this kind. As to animal foods, when normal, they are usually undisturbed by man, and always answer to the chemical tests.

[TO BE CONTINUED.]

